# Prostate-specific membrane antigen-targeting radiopharmaceuticals: a new frontier in hepatic malignancies

**DOI:** 10.3389/fonc.2025.1547459

**Published:** 2025-03-07

**Authors:** Fucen Liu, Liming Xiao, Ling Zhao, Yi Tao, Dan Huang, Zhengguo Chen, Chuandong He, Chunyan Wu

**Affiliations:** ^1^ National Health Commission (NHC) Key Laboratory of Nuclear Technology Medical Transformation (MIANYANG CENTRAL HOSPITAL), Mianyang, China; ^2^ Department of Nuclear Medicine, Mianyang Central Hospital, School of Medicine, University of Electronic Science and Technology of China, Mianyang, China; ^3^ Institute of Basic Medicine, North Sichuan Medical College, Nanchong, China

**Keywords:** prostate-specific membrane antigen, hepatic malignant tumors, radiopharmaceuticals, imaging, therapy

## Abstract

**Background/Objectives:**

Prostate-specific membrane antigen (PSMA) is overexpressed in prostate hypercellularity, making it an effective target for molecular imaging and therapy of prostate cancer. PSMA is expressed in the neovasculature of hepatic malignancies and regulates tumor cell invasion and angiogenesis. The diagnosis and treatment of hepatic malignancies remain challenging. Thus, radiopharmaceuticals targeting PSMA are gaining prominence in the treatment of hepatic malignancies. Therefore, this review aims to discuss the applications of PSMA-targeting radiopharmaceuticals in hepatic malignant tumors, focusing on hepatocellular carcinoma (HCC), to assess their value as a diagnostic and therapeutic agent for hepatic malignancies.

**Methods:**

The potentials of PSMA-targeting radiopharmaceuticals for diagnostic and therapeutic use in hepatic malignancies were investigated. Moreover, their characteristics, diagnostic and therapeutic efficacies, and potential synergies when used in conjunction with other therapeutic modalities were elucidated.

**Results:**

Computed tomography (CT) and magnetic resonance imaging (MRI) are the most common imaging modalities in clinical practice; however, their sensitivity is not optimal. PSMA positron emission tomography/CT can be used as a complementary modality to conventional imaging for characterizing lesions, staging and/or re-staging HCC, and assessing treatment response when conventional imaging results are unclear. Moreover, most patients with HCC are diagnosed at an advanced stage in which treatment options are limited. Hence, PSMA-based radioligand therapy serves as a promising alternative treatment when multiple treatments fail.

**Conclusions:**

Further research and clinical transformation are required to effectively diagnose and treat HCC via PSMA targeting. This will have significant clinical application prospects in primary and secondary hepatic malignancies.

## 1 Introduction

Hepatocellular carcinoma (HCC), a highly vascularized tumor, is the most common type of primary hepatic malignancy and the third most common cause of cancer-related deaths worldwide (1, 2). Angiogenesis is a common feature of solid tumors and is responsible for tumor growth, invasion, and metastasis (3). Abnormally proliferated blood vessels provide nutrients for HCC development and promote tumor growth, with a high tendency of local, regional, or distant metastasis (4, 5). The liver receives double blood supply from the portal vein and hepatic artery, making it a blood-rich organ. Therefore, malignant tumors in other organs can be transferred to the liver through the blood route, where they continue to grow and spread, forming secondary hepatic malignant tumors.

HCC and secondary hepatic malignant tumors are primarily diagnosed using computed tomography (CT) or magnetic resonance imaging (MRI) in line with the Liver Imaging and Reporting Data System criteria (6). This is supplemented with a combination of serum alpha-fetoprotein (AFP) and certain tumor markers. However, diagnosing patients with small lesions, poor blood supply, and lack of abnormal serum AFP and tumor markers remains clinically challenging, and conventional imaging based on morphology cannot effectively judge the systemic invasion and biological activity of tumors.

Positron emission tomography (PET), an imaging modality that evaluates disease by labeling molecular targets with radionuclides, is often combined with CT or MRI to form multi-modal imaging of PET/CT or PET/MRI to improve diagnostic efficiency (7). Fluorine-18 fluorodeoxyglucose ([^18^F]FDG), a glucose analogue, is the most commonly used PET radiotracer (8). Moreover, [^18^F]FDG PET/CT is valuable in staging, treatment guidance, efficacy evaluation, and prognosis prediction of hepatic malignancies; however, its low diagnostic accuracy for well-differentiated hepatic malignancies limits its application. Well-differentiated hepatic malignancies have enzymatic activities similar to those of normal hepatocytes and tend to have higher glucose-6-phosphatase activity, resulting in dephosphorylation of intracellular FDG and expulsion from the cell. Therefore, new PET imaging agents that are more suitable for diagnosing hepatic malignant tumors remain warranted.

Prostate-specific membrane antigen (PSMA) is a type II transmembrane glycoprotein encoded by the folate hydrolase 1 (FOLH1) gene, which is highly expressed in prostate cancer cells. However, PSMA is overexpressed in neovascular endothelial cells and other non-prostate cancer tumors (HCC, glioblastoma, renal carcinoma, lung, ovarian, breast, thyroid, salivary adenocarcinoma, melanoma, and mesothelioma). PSMA expression is upregulated in neovascular endothelial cells of more than 70% of solid tumors, with HCC and medullary thyroid carcinomas most frequently expressing PSMA in neovasculature among primary tumors. Interestingly, although most solid cancers do not express PSMA on the tumor cells, it is still observed in salivary gland tumors (especially adenoid cystic carcinoma, up to 91%), and to a lesser extent in hepatocellular, lung, and breast cancer tissues (9–11). In addition, preclinical findings suggest that PSMA regulates tumor cell invasion and angiogenesis by degrading the extracellular matrix and regulating the integrin signaling of endothelial cells (12, 13). Therefore, radioligand-based diagnosis and treatment targeting PSMA is not limited to prostate cancer (14), but may also be useful for the screening, targeting, and efficacy monitoring of patients with other anti-angiogenic therapies.

High PSMA expression levels have been reported in tumor neovasculature (89.9%) and tubular membranes of tumor cells (41.1%) in most patients with HCC (15, 16), which correlate with poor prognosis (17). Accordingly, PSMA expression in HCC neovasculature is fundamental for PSMA-targeting radiopharmaceuticals for imaging and therapy (18), which will facilitate new diagnosis and treatment modalities for HCC. Therefore, this review aims to provide a comprehensive overview of the research state of PSMA-targeting radiopharmaceuticals in hepatic malignancies. It discusses the potential of PSMA-targeting radiopharmaceuticals for diagnostic and therapeutic use in hepatic malignancies and elucidates their characteristics, diagnostic and therapeutic efficacies, and potential synergies when used in conjunction with other therapeutic modalities.

## 2 Expression characteristics of PSMA in primary hepatic malignant tumors and their clinicopathological relationship

PSMA is expressed in the blood vessels associated with several non-prostate solid tumors. Tolkach et al. (15) investigated an immunohistochemical cohort of 153 patients with HCC, where PSMA was identified in tumor tissues in both tubular membrane (41.1%) and neovascularization (89.9%) expression patterns. Only 4.1% of tumors were completely negative for both patterns, and 79.2% of tumors exhibited elevated PSMA expression at any site. In addition, Chen et al. (19) reported that 13 of 15 patients with primary cholangiocarcinoma (86.7%) and 15 of 22 patients with HCC (68.2%) showed PSMA vascular expression. Out of 22 HCC cases, the expression of PSMA was easier to recognize in grade 3 HCC than in grade 2; however, PSMA was not expressed in fibrolamellar HCC, normal liver tissue, or non-neoplastic cirrhosis. The expression of PSMA is considerably higher in cirrhotic HCC than in non-cirrhotic HCC and in patients with HCC and hepatitis B or C virus infection than in non-viral infection cohorts. Moreover, Chen et al. (20) reported that 161 of 203 patients with cholangiocarcinoma (79.3%) and 185 of 213 patients with HCC (86.8%) showed positive PSMA expression, mainly in the neovascular endothelial cells surrounding the tumor. The expression of PSMA was positively correlated with the stage and grade of HCC, and the positive rate of PSMA was higher in patients with high-grade and advanced stages. However, no link was found between PSMA expression and several variables, including sex, age, region, AFP, hepatitis B surface antigen, and tumor size. Similarly, Jiao et al. (17) showed that 76 of 103 (73.8%) patients with HCC exhibited positive expression of PSMA, of which 27 were positive for PSMA in more than 50% of tumor-associated vessels and 49 in less than 50% of tumor-associated vessels. PSMA expression correlates with tumor stage, degree of differentiation, lymph node metastasis, Ki-67 index, and other clinicopathological features; high vascular PSMA expression is an indicator of poor prognosis in patients with HCC. Patients with HCC benefit from PSMA as an independent prognostic marker and vascular therapeutic target. Kmeid et al. (21) demonstrated that 55 of 68 (80.9%) patients with HCC showed positive PSMA expression, thereby confirming PSMA as a neovascular marker. PSMA expression is more specific and accurate than cluster of differentiation (CD)34 expression. Hence, compared with CD34, PSMA better distinguishes HCC from benign and precursor hepatic lesions. Kunikowska et al. (16) observed that PSMA expression in tumor-associated vessels exhibits notable heterogeneity in intensity and distribution. The HCC pseudo glandular phenotype is weakly expressed and observed only in a few vessels, whereas the HCC trabecular phenotype is strongly expressed in vessels. Furthermore, Li et al. (22) reported that the positive expression of PSMA in HCC is linked to advanced tumor stage and grade, an independent risk factor for poor disease survival and a potential diagnostic and prognostic biomarker of HCC. These findings align with those of Chen et al. (20) and Jiao et al. (17). The authors reported that HCC with positive PSMA expression is more likely to be positive for programmed death ligand 1, thereby reinforcing the notion that these two proteins interact in regulating the tumor microenvironment. Among the above studies, the study by Chen et al. (19) reported PSMA expression as a qualitative variable, whereas the other studies (15–17, 20–22) reported it as a quantitative variable.

## 3 Research on PSMA PET imaging

### 3.1 Research on PSMA PET imaging of primary hepatic malignant tumors

Many case reports and a few prospective studies have investigated molecular imaging targeting PSMA in primary hepatic malignancies, with HCC being the most common. **Table 1** (15, 23–42) briefly summarizes the characteristics and results of PSMA PET in the diagnosis of primary hepatic malignancies of case reports. In most case reports, PSMA PET demonstrates superior diagnostic efficacy compared to FDG PET, exhibiting enhanced diagnostic performance, which substantiates the utility of PSMA-targeted molecular imaging in evaluating primary hepatic malignancies. Ozkan et al. (43) reported a case of a patient with HCC at 2 months after yttrium-90 resin microsphere treatment in which a definite response to treatment was observed at [^68^Ga]Ga-PSMA PET/MR. This finding suggests the possibility of using [^68^Ga]Ga-PSMA PET to evaluate the therapy response of patients with HCC.

**Table 1:** Results of the literature review on PSMA PET imaging of primary hepatic malignancy.

	Reference (First author)	Year	Age/Sex	PSMA PET	FDG PET	Other malignancy
HCC	Sasikumar (23)	2016	78/M	Intense tracer accumulation in the liver lesion with mild tracer uptake in the bone lesions and lung lesions	N/A	N/A
HCC	Taneja (24)	2017	77/M	A PSMA-avid hepatic lesion	N/A	Prostate cancer
HCC	Patel (25)	2017	66/M	PSMA-avid foci in segments 2, 7, and 8 of the liver	Not FDG avid	Prostate cancer
HCC	Huang (26)	2018	66/M	Intensely tracer-avid nodule in segment VII of the liver	N/A	Prostate cancer
HCC	Perez (27)	2019	87/M	Focal and intense uptake of radiotracer in a different region of the tumor	Minimal FDG uptake, slightly above the liver background	Melanoma
HCC	Tolkach (15)	2019	62/M	Intense and heterogeneous PSMA uptake	N/A	N/A
HCC	Das (28)	2019	69/M	With increased PSMA expression at the periphery of the lesion	N/A	Prostate cancer
HCC	Erhamamcı (29)	2020	69/M	High PSMA uptake in both the metastatic bone lesions and the primary liver lesion	Mild FDG uptake in the bone lesions, but no FDG uptake in the liver lesion	N/A
HCC	Zhao (30)	2020	77/M	High tracer uptake in the primary lesion in the liver and the peripheral zone of the prostate	N/A	Prostate cancer
HCC	Erhamamcı (31)	2020	74/M	Intense PSMA uptake in the lesion	Slight FDG uptake with heterogenous character	N/A
HCC	Muzaffar (32)	2021	45/M	Heterogenous increase in tracer uptake in segment VII of the liver	No abnormal FDG localization in the liver	Colorectal cancer
HCC	Weitzer (33)	2021	69/M	Significant pathological uptake in the right liver lobe	No abnormal uptake in the liver	Prostate cancer, esophageal adenocarcinoma
HCC	Usmani (34)	2021	82/M	High PSMA-expressing hepatic lesions (the primary tumor), additional multifocal hepatic lesions, and multiple bone metastases	N/A	N/A
HCC	Wang (35)	2023	78/M	Abnormal radioactive uptake	Radioactive uptake similar to the surrounding normal liver	N/A
HCC	Hekman (36)	2023	76/M	Tracer-avid lesion in the left lobe of the liver	N/A	Bilateral RCC, prostate cancer
HCC	Reiter (37)	2024	70/M	Moderate uptake in the liver	N/A	Prostate cancer
ICC	Chahinian (38)	2020	79/M	Focal radiotracer uptake in segments VI and VII of the liver	N/A	Non-Hodgkin lymphoma, prostate cancer
ICC	Sun (39)	2024	71/M	Abnormally increased radiotracer uptake in the liver mass	Low FDG uptake	Prostate cancer
CHC	Alipour (40)	2017	70/M	Multiple hepatic foci of avid PSMA uptake in both lobes of the liver	N/A	N/A
CHC	Kang (41)	2022	69/M	Intensely PSMA–avid focus in liver segment 8	Moderately FDG-avid focus in liver segment 8	Prostate cancer
CHC	Chalikandy (42)	2023	62/M	Non-PSMA-expressing hypodense liver lesions involving segment VII/VIII	FDG-avid hypodense liver lesions involving segment VII/VIII	Prostate cancer

PSMA, prostate-specific membrane antigen; FDG, fluorodeoxyglucose; HCC, hepatocellular carcinoma; M, male; N/A, not available; PSA, prostate-specific antigen; ICC, intrahepatic cholangiocarcinoma; CHC, combined hepatocellular-cholangiocarcinoma.

In addition, a few prospective studies have confirmed the affinity of PSMA for HCC, and the clinical feasibility of PSMA PET for HCC imaging is supported by a higher level of evidence-based medical evidence. Lu et al. (44) demonstrated that [^68^Ga]Ga-PSMA PET was a promising imaging modality for HCC using different types of HCC xenograft models. Furthermore, Kunikowska et al. (16) demonstrated that [^68^Ga]Ga-PSMA PET/CT was effective for detecting PSMA expression in patients with HCC, and there was no significant difference between patients with newly diagnosed HCC and those with recurrent HCC in terms of [^68^Ga]Ga-PSMA uptake. Comparison with CT or MRI showed the presence of PSMA uptake in the enhanced part of the tumor but not in the necrotic part, and no significant correlation was observed between the concentrations of serum tumor markers (AFP, CA19-9, and CEA) and [^68^Ga]Ga-PSMA PET parameters. In addition, [^68^Ga]Ga-PSMA PET/CT showed more lesions in the liver than that shown by CT or MRI, leading to changes in the initial treatment plan. However, Hirmas et al. (45) reported that [^68^Ga]Ga-PSMA PET and CT had comparable accuracies for staging at the liver level, whereas [^68^Ga]Ga-PSMA PET performed better at the extrahepatic level, demonstrating higher accuracy than that of CT in detecting HCC metastases. Moreover, Wong et al. (46) observed that PSMA PET/CT was as sensitive as MRI in detecting HCC, with sensitivities of 91% and 87% for PSMA PET/CT and MRI, respectively, with specificities of 70% and 73%, respectively, and a high negative predictive value (90%) for PSMA PET/CT. In addition, Shamim et al. (47) suggested [^68^Ga]Ga-PSMA PET/CT as a supplement to conventional imaging for the diagnosis, staging, and re-staging of advanced HCC and treatment response evaluations. They observed no correlation between the maximum standardized uptake value (SUVmax) of the [^68^Ga]Ga -PSMA PET/CT tumor and the AFP levels. Hence, the study concluded that patients with SUVmax > 10 in lesions on [^68^Ga]Ga-PSMA PET/CT were potential candidates for [^177^Lu]Lu-PSMA RLT and required further investigation. Kesler et al. (48) demonstrated that [^68^Ga]Ga-PSMA PET/CT was superior to [^18^F]FDG PET/CT in terms of imaging patients with HCC. Out of the 37 HCC lesions, 36 showed a positive uptake of [^68^Ga]Ga-PSMA, only 10 showed positive uptake of [^18^F]FDG, and four hepatic regenerative nodules showed negative uptake on both [^68^Ga]Ga-PSMA PET/CT and [^18^F]FDG PET/CT. This result may be ascribed to the fact that branches of the hepatic artery mainly supply HCC. By contrast, regenerative and dysplastic nodules are primarily supplied by the portal vein. Kesler et al. (48) further compared the uptake of [^68^Ga]Ga-PSMA with the results of enhanced CT. A close correlation was observed between the uptake of [^68^Ga]Ga-PSMA and the distribution of lesion vessels expressed on enhanced CT, with a considerable difference between the uptake of [^68^Ga]Ga-PSMA in enhanced and non-enhanced lesions. Uptake of [^68^Ga]Ga-PSMA is higher in enhanced lesions, whereas [^18^F]FDG uptake is higher in non-enhanced lesions, which aligns with the results of Kunikowska et al. (16). Gündoğan et al. (49) confirmed that [^18^F]FDG PET/CT is less effective than [^68^Ga]Ga-PSMA PET/CT for staging of HCC, and a high uptake of [^68^Ga]Ga-PSMA can be used for targeting PSMA RLT. They also confirmed no correlation between [^68^Ga]Ga-PSMA tumor uptake and serum AFP levels, indicating that tumor angiogenesis and AFP production are independent in HCC. However, Kuyumcu et al. (50) reported less optimistic results that advanced HCC could be evaluated using [^68^Ga]Ga-PSMA PET, but it was not superior to [^18^F]FDG PET. In addition, a moderate correlation was observed between the SUVmax of [^68^Ga]Ga-PSMAPET/CT and overall survival.

Two meta-analyses have also quantified PSMA-targeting radiopharmaceuticals for HCC, confirming that PSMA PET is a promising imaging modality for diagnosing and staging HCC. One meta-analysis of six selected studies (126 patients with HCC) provided a DR of 85.9% for [^68^Ga]Ga-PSMA-11 PET/CT and PET/MRI in the diagnosis of HCC. Therefore, the quantitative data provided demonstrate the high DR of PET/CT or PET/MRI with PSMA-targeting radiopharmaceuticals for HCC lesion detection. Because one of the included studies involved PET/MR, moderate statistical heterogeneity was found in the included studies (I^2^ = 56%) (51). Another meta-analysis of nine selected studies (196 patients with HCC and 491 HCC lesions) demonstrated that [^68^Ga]Ga-PSMA-11 PET showed a high sensitivity of 89.9% (95% CI 78.5-95.5) on a per-patient analysis for HCC and that this rate increased to 94.5% (95% CI 82.9-98.4) on a per-lesion analysis (52). However, the existing literature does not provide enough data to confidently evaluate its specificity and accuracy.

### 3.2 Research on PSMA PET imaging of secondary hepatic malignant tumors

PSMA PET imaging of secondary hepatic malignancies, in addition to primary hepatic malignancies, has also been reported. **Table 2** (53–58) summarizes the results and characteristics of case reports on PSMA PET imaging of secondary hepatic malignancies. Two retrospective studies with larger sample sizes analyzed [^68^Ga]Ga-PSMA PET for liver metastases in patients with prostate cancer. Damjanovic et al. (59) detected 103 liver metastases in 18 out of 739 patients with prostate cancer, 80 of which were PSMA positive and 23 PSMA negative. The SUVmax in PSMA-positive liver metastases was markedly higher than that in normal liver tissues, whereas the SUVmax in PSMA-negative liver metastases was lower than that in normal liver tissues. Moreover, most prostate cancer liver metastases exhibit high PSMA expression in [^68^Ga]Ga-PSMA PET imaging. However, the possibility of PSMA-negative expression in certain liver metastases remains. Mattoni et al. (60) reported that the sensitivity, specificity, positive predictive value, negative predictive value, and accuracy of PSMA PET in diagnosing liver metastasis were 0.58, 0.92, 0.82, 0.77, and 0.78, respectively. The area under the curve of the radiation characteristic model combined with sphericity and moment of contrast in multiple regression analysis was 0.807. Furthermore, [^68^Ga]Ga-PSMA PET for diagnosing liver metastases exhibits moderate sensitivity, higher specificity, higher positive predictive value, and higher repeatability than conventional imaging (CT or MRI) and liver biopsy.

**Table 2:** Characteristics of PSMA PET imaging of secondary hepatic malignancy.

Reference(First author)	Year	Age/Sex	PSMA PET	FDG PET	Malignancy
Marafi (53)	2019	75/M	Multiple focal areas of increased uptake at both lobes of the liver	Increased FDG uptake at hepatic segment VII	Cholangiocarcinoma
Önner (54)	2022	75/M	Solitary PSMA–avid hypodense lesion in liver segment 7	N/A	Prostate cancer
Chen (55)	2023	53/M	Increased uptake in a nodule in the left lobe of the liver	Not FDG avid	Pancreatic neuroendocrine tumor (G2)
Doroudinia (56)	2023	74/M	Several PSMA-avid lesions in the liver	no FDG-avid lesion.	Colon cancer (primary), prostate cancer
Winter (57)	2023	N/A/M	Intense PSMA uptake in the liver	N/A	Duodenal neuroendocrine tumor (primary), prostate cancer
Stanzel (58)	2023	88/M	Extensive PSMA-negative lesions in the liver segments 3, 4, 5, and 8	N/A	Prostate cancer

PSMA, prostate-specific membrane antigen; FDG, fluorodeoxyglucose; M, male; N/A, not available.

### 3.3 Research on PSMA PET imaging of benign liver lesions

Positive expression of PSMA PET imaging can be observed in various benign liver lesions, including post-radiotherapy inflammatory injury, focal inflammation, fatty infiltration, hemangioma, focal nodular hyperplasia of the liver, and abnormal perfusion. The characteristics of PSMA PET imaging of these benign lesions are summarized in **Table 3** (60–68). Available studies on PSMA PET imaging of benign lesions mainly focus on case reports, and systematic studies with large sample sizes are lacking.

**Table 3:** Characteristics of PSMA PET imaging of benign liver lesions.

Reference(First author)	Year	Age/Sex	PSMA PET	FDG PET	Malignancy	Final Diagnosis
Bhardwaj (61)	2016	77/M	Intense PSMA uptake in hepatic segment IVa	N/A	Prostate cancer	Liver hemangioma
Ladrón-de-Guevara (62)	2017	68/M	Three rounded liver intense uptake foci	N/A	Prostate cancer	Inflammation
Hoberück (63)	2020	80/M	Intensive PSMA expression in segment II of the liver	N/A	Prostate cancer	Hepatic Vascular Malformation
Bilgic (64)	2021	66/M	Intense uptake in liver segment VI	N/A	Prostate cancer	Focal nodular hyperplasia
Mattoni (60)	2022	N/A/M	Positive PSMA uptake	N/A	Prostate cancer	Focal hepatitis and perfusion defect
Saad (65)	2022	64/M	Wedge-shaped area of increased PSMA avidity in segments V and VIII of the liver	N/A	Prostate cancer	Fatty infiltration
Adediran (66)	2023	78/F	Band-like area of increased radiotracer uptake	[^18^F]fluciclovine PET: photopenia in the corresponding area	Breast carcinoma	Radiation-induced hepatitis
Silveira (67)	2024	62/M	Regional geographic tracer avidity was seen in the midline left hepatic lobe	FDG PET: no FDG avidity	Prostate cancer	Inflammatory related to radiation
Alsaleh (68)	2024	51/M	Focal PSMA-avid liver lesion	N/A	Prostate cancer	Liver hemangioma

PSMA, prostate-specific membrane antigen; FDG, fluorodeoxyglucose; F, female; M, male; N/A, not available.

## 4 PSMA RLT

The aforementioned studies and case reports revealed the expression of PSMA in primary and secondary liver malignant tumors. Studies have shown the therapeutic feasibility and efficacy of targeted PSMA radioligand therapy in the liver showing PSMA-positive expression. For primary HCC, in a PSMA-positive HCC xenograft mouse model, Lu et al. (69) conducted *in vivo* RLT using [^177^Lu]Lu-PSMA-617 and [^177^Lu]Lu-EB-PSMA-617. RLT with [^177^Lu]Lu-PSMA-617 and [^177^Lu]Lu-EB-PSMA-617 markedly impedes tumor growth and extends survival time without notable toxicity. The higher tumor uptake and longer blood persistence of [^177^Lu]Lu-EB-PSMA-617 than [^177^Lu]Lu-PSMA-617 confirm the promising clinical use of these radioligands. However, some clinical studies have shown less-promising results. In the study cohort by Hirmas et al. (45), two patients with HCC with high [^68^Ga]Ga-PSMA PET uptake were treated with [^177^Lu]Lu-PSMA-617. SPECT/CT-based dosimetry during treatment showed that the tumor radiation dose from RLT was 10 times less than that received from conventional one-cycle external beam radiotherapy for HCC, which is not as effective. Hence, RLT was terminated in these two patients after one cycle. Similarly, Pretet et al. (70) reported a case of a patient with HCC treated with [^177^Lu]Lu-PSMA radiation therapy that did not achieve the desired outcome. The patient was diagnosed with castration-resistant prostate cancer (CRPC) with alcoholic cirrhosis and multiple HCC nodules. The [^68^Ga]Ga-PSMA PET/CT revealed high PSMA expression in prostate and HCC lesions. However, two post-treatment evaluations of [^177^Lu]Lu-PSMA demonstrated efficacy only in bone metastases of prostate cancer, whereas HCC showed progressive changes.

Encouraging results have been achieved by PSMA-targeting RLT in secondary hepatic malignancies. Wei et al. (71) reported a patient with CRPC with multiple lymph nodes and bone and liver metastases, in which the size of all metastases and PSMA expression remarkably subsided following treatment with [^177^Lu]Lu-PSMA-617. Moreover, Khreish et al. (72) confirmed that [^177^Lu]Lu-PSMA-617 RLT could control liver metastases, resulting in prolonged progression-free survival and pronounced improvement in overall survival. This finding supports the application of [^177^Lu]Lu-PSMA-617 therapy in advanced/terminal CRPC with liver metastases.

## 5 Discussion

PSMA is a type II transmembrane glycoprotein initially found in benign prostate epithelium. The protein is upregulated in high-grade and advanced prostate cancer and supports angiogenesis and promotes cancer cell migration (12, 73, 74). The expression and function of PSMA have received increasing attention in other types of malignancies. Non-prostate cancer cell lines express PSMA at nearly 30 times lower levels than PSMA-positive prostate cells (LNCaP cells) (75). However, PSMA is overexpressed in neoplastic microvessels of non-prostate tumors, and more than 70% of tumor-associated vessels of HCC exhibit high PSMA levels (9–11). Therefore, PSMA is a new potential target for HCC diagnosis and treatment (15, 76–78).

PSMA is undetected in the vasculature of normal liver tissue (15, 17, 48), and it is specifically expressed in the neovascular endothelium associated with HCC tumors (16). However, considerable differences in positivity rates have been reported (15, 17, 19, 79). Jiao et al. (17) and Chen et al. (19) confirmed that PSMA expression is associated with HCC stage and grade. Patients with high tumor stage and heavy lymph node metastasis load are more likely to exhibit PSMA overexpression, and those with grade 3 HCC are more likely than those with grade 2 HCC to have peritumoral/vascular expression. In addition, PSMA peritumoral/vascular expression is markedly higher in cirrhotic than in non-cirrhotic HCC and markedly higher in viral hepatitis-infected than in non-infected individuals. PSMA expression in HCC is heterogeneous and elevated in inflammatory environments and cirrhosis; however, the underlying mechanisms remain to be elucidated.

Imaging has become the preferred method for confirming the diagnosis of hepatic malignancy because of the inherent difficulties of pathology. These include the risk of bleeding, the challenge of accurately targeting the tumor, and the potential for cancer cell dissemination. MRI and CT are the most common imaging modalities in clinical practice, but their sensitivities are poor (16, 80), and small lesions are challenging to detect, particularly in patients with cirrhosis. Nuclear medicine molecular probe imaging facilitates early diagnosis of diseases at the molecular level; however, currently available imaging agents have limitations. Thus, better-targeted molecular probes are necessary to diagnose tumors early.

The advantages of PSMA PET/CT in diagnosing HCC have been confirmed, with sensitivity comparable with that of MRI and superior to that of CT in detecting primary foci of HCC, with ratios of 91% vs. 87% vs. 32% (45, 46). The [^18^F]FDG PET/CT is the most common broad-spectrum tumor imaging modality for evaluating HCC with or without lymph nodes and distant metastases. However, its low sensitivity to primary foci of moderately and highly differentiated HCC and its susceptibility to false-negative results (81) limit its applications in HCC. PSMA PET/CT demonstrated a greater number of lesions that [^18^F]FDG PET/CT, with higher uptake and tumor-background ratio (48, 49).

Therefore, PSMA PET/CT can be used as a complementary modality to conventional imaging for characterizing lesions, staging and/or re-staging HCC, and assessing treatment response when conventional imaging results are unclear, thereby contributing to early diagnosis and improved HCC treatment (47). In secondary hepatic malignancies, most of the liver metastases of prostate cancer have high PSMA expression; hence, they can be detected using PSMA PET/CT. Negative or low PSMA expression may be a result of disease progression presenting as neuroendocrine differentiation. In addition, liver metastases secondary to other tumors, such as colon cancer and neuroendocrine tumors, have been detected using PSMA PET/CT (38, 39, 41, 56, 57).

PSMA PET/CT may yield false positive results. The observation of PSMA expression in acute and chronic inflammation may be attributed to increased local blood flow, vascular permeability, and folate receptors in macrophages expressing PSMA. In addition to inflammation, high PSMA expression in other non-neoplastic lesions, such as vascular disease (vascular malformations/hemangiomas), focal nodular hyperplasia of the liver, and focal fatty liver, should be considered when assessing hepatic parenchymal lesions using PSMA PET (61, 63–65, 68). These lesions may be accompanied by changes in local blood flow and metabolism, with a more complex vascular network, faster blood flow, and more vigorous metabolic activity, resulting in increased radiotracer uptake by PSMA PET/CT. Moreover, inflammatory or immune reactions may accompany hemangiomas/vascular malformations, and infiltration of inflammatory cells, immune cells, or other cell types may alter the local metabolic environment of the tissue. This phenomenon will affect the distribution and uptake of the tracers. In addition, hepatic perfusion abnormalities suggested by enhanced CT are sometimes highly expressed in PSMA PET/CT (60) imaging. This is possible because pathological processes, such as alterations in local hepatic perfusion, inflammation, or tumors, are often accompanied by increased blood supply and metabolism, thereby increasing radiotracer uptake. Accordingly, liver lesions with a positive PSMA PET/CT test may not be HCC or liver metastases and should be considered in combination with clinical information and other diagnostic methods.

Most patients with HCC are diagnosed at an advanced stage in which treatment options are limited. PSMA RLT may be considered a palliative alternative treatment when multiple treatments fail. First-line treatment of unresectable and metastatic HCC is a combination of anti-angiogenic therapy and immunotherapy, utilizing the agents bevacizumab and atezolizumab (82). PSMA PET/CT allows for screening patients eligible for anti-angiogenic therapy and monitoring of treatment. PSMA is ex-pressed in HCC tumor-associated vessels, which are effective targets for anti-angiogenic therapy (83). The treatment of [^177^Lu]Lu-PSMA in other PSMA-expressing tumors (glioblastoma and renal clear cell carcinoma) has suggested the possibility of this targeted therapy (84–86). In addition, other radionuclide treatments have been used for HCC before PSMA RLT. Transarterial injection of radiopharmaceuticals with yttrium-90-labeled microspheres has been approved by the US Food and Drug Administration for patients with HCC and unresectable masses up to 8 cm in diameter, and personalized dosimetry of this modality enhances treatment response (87). Additionally, radioactive iodine (^131^I)-labeled metuximab targeting CD147 can be used as a systemic radiotherapeutic therapy for HCC (88) and has been successfully combined with transcatheter hepatic arterial chemoembolization for unresectable HCC, thereby improving overall survival (89). Therefore, PSMA RLT can be similarly applied to advanced HCC and, unlike other liver-directed therapies, used for systemic treatment via blood transfusion in metastatic HCC.

A preclinical study reported that [^177^Lu]Lu-PSMA RLT pronouncedly inhibits tumor growth and prolongs survival time without obvious toxicity in PSMA-positive HCC xenograft mice, thereby providing a basis for future research on [^177^Lu]Lu-PSMA RLT HCC (69). However, a small number of clinical studies have shown less favorable results. Patients with HCC may need to meet certain conditions to benefit from PSMA RLT. In accordance with the European Association for Nuclear Medicine guidelines pertaining to the pilot Phase II trial of [^177^Lu]Lu-PSMA-617, in [^68^Ga]Ga-PSMA-11 PET/CT, the SUVmax of the tumor lesion must be at least 1.5 times higher than the liver baseline SUVmean to qualify for treatment (83, 90). Moreover, patients with lesions with SUVmax > 10 on PSMA PET/CT are potential candidates for [^177^Lu]Lu-PSMA treatment (45). Future studies should focus on how to screen for patients with HCC who will benefit from definitive studies. Despite the negative clinical reports available (45, 70), large-sample prospective studies remain warranted to confirm the value of PSMA RLT in HCC treatment, the potential synergistic effects of combining other therapeutic modalities, and the development of individualized treatment regimens based on the patient’s genotype, staging, and other information. Positive results have been reported on PSMA RLT in secondary hepatic malignancies, especially in patients with metastatic CRPC, which can be effectively treated with [^177^Lu]Lu-PSMA RLT. This results in a near-complete remission of hepatic metastases and prolongs the overall survival of patients without hematological and hepatic or renal toxicity (71, 72).

To date, most of the published literature on targeted PSMA radiopharmaceuticals for hepatic malignancies are small studies and case reports, and large prospective studies are rare. Patients with high PSMA uptake are likely reported, while negative outcomes are less likely to be published. However, it is precisely because the current limited data provide novel and effective targets for the future diagnosis and treatment of hepatic malignant tumors, and we have reason to believe that PSMA-based radionuclide imaging and therapy will be applied to hepatic malignant tumors.

## 6 Conclusion

In this review, we outlined the state of research on radionuclide-labeled PSMA radiopharmaceuticals in liver lesions and discussed their potential applications. The expression of PSMA in the neovascularization system of HCC tumors has been confirmed, and its expression in HCC is notably heterogeneous. Numerous studies and case reports have confirmed the potential of PSMA PET/CT as an alternative imaging modality to conventional imaging in primary and secondary hepatic malignancies. However, potential false positives or negatives remain to be addressed. Despite the unsatisfactory results of PSMA RLT in HCC treatment, given the lack of effective therapeutic options for patients with advanced HCC, large-sample prospective studies remain warranted to validate its therapeutic value and potential synergistic effects when combined with other therapeutic modalities. With further research and clinical transformation, diagnosing and treating HCC via PSMA targeting will have significant clinical application prospects in primary and secondary hepatic malignancies.
